# Preliminary results of the acute *H*eart *F*ailure registry in the *DELTA* region of Egypt (DELTA-HF): a database and a quality initiative project

**DOI:** 10.1186/s43044-019-0024-0

**Published:** 2019-11-26

**Authors:** Abdelfatah Elasfar, Sherif Shaheen, Wafaa El-Sherbeny, Hatem Elsokkary, Suzan Elhefnawy, Mohammed Al-Setiha

**Affiliations:** 1 Department of Cardiovascular Medicine, Faculty of Medicine, Tanta University, Tanta, Egypt; 2 Madinah Cardiac Center, Madinah, Saudi Arabia

**Keywords:** Acute heart failure, Egypt, Registry, Quality initiative

## Abstract

**Background:**

Data about heart failure in Egypt is scarce. We aimed to describe the clinical characteristics and diagnostic and treatment options in patients with acute heart failure in the Delta region of Egypt and to explore the gap in the management in comparison to the international guidelines.

**Results:**

DELTA-HF is a prospective observational cohort registry for all consecutive patients with acute heart failure (AHF) who were admitted to three tertiary care cardiac centers distributed in the Delta region of Egypt. All patients were recruited in the period from April 2017 to May 2018, during which, data were collected and short-term follow-up was done.

A total of 220 patients (65.5% were males with a median age of 61.5 years and 50.9% had acute decompensation on top of chronic heart failure) was enrolled in our registry. The risk factors for heart failure included rheumatic valvular heart disease (10.9%), smoking (65.3%), hypertension (48.2%), diabetes mellitus (42.7%), and coronary artery disease (28.2%). Left ventricular ejection fraction (LVEF) was less than 40% in 62.6%. Etiologies of heart failure included ischemic heart disease (58.1%), valvular heart disease (16.3%), systemic hypertension (9.1%), and dilated non-ischemic cardiomyopathy (15.5%). Exacerbating factors included infections (28.1%), acute coronary syndromes (25.5%), non-compliance to HF medications (19.6%), and non-compliance to diet (23.2%) in acute decompensated heart failure (ADHF) patients. None of our patients had been offered heart failure device therapy and only 50% were put on beta-blockers upon discharge. In-hospital, 30 days and 90 days all-cause mortality were 18.2%, 20.7%, and 26% respectively.

**Conclusions:**

There is a clear gap in the management of patients with acute heart failure in the Delta region of Egypt with confirmed under-utilization of heart failure device therapy and under-prescription of guideline-directed medical therapies particularly beta-blockers. The short-term mortality is high if compared with Western and other local registries. This could be attributed mainly to the low-resource health care system in this region and the lack of formal heart failure management programs.

## Background

Heart failure (HF) is a global public health problem affecting an estimated 26 million people worldwide [1]. In the USA, heart failure currently results in more than one million hospitalizations every year and the estimated 5-year mortality is approximately 50% [2]. The burden of heart failure is projected to increase over the next decade, and it is predicted that 1 in every 33 Americans will be affected by heart failure [3]. Acute HF represents a period during which the likelihood of death, complications, and re-hospitalization is significantly greater than in a comparable period of chronic but stable HF [4]. The overall number of hospitalizations for heart failure continues to grow as a consequence of the aging of the population, improved survival after acute myocardial infarction, and effective prevention of sudden cardiac death [5, 6]. For many years, data about heart failure was only restricted to Western countries. In the last few years, new emerging data about heart failure came from some countries in the Middle East region including HEARTS and Gulf CARE registries [7, 8]. In Egypt, data about heart failure was very scarce till very recently when Egypt was included in the European heart failure survey about both acute and chronic heart failure [9]. In this study, we sought to report the baseline clinical characteristics, management, and short-term outcome for acute heart failure (AHF) patients in three tertiary care centers in the Delta region of Egypt and to explore the gap in the management in comparison to the international recommendations.

## Methods

### Study design

DELTA-HF is a prospective cohort registry for all consecutive patients with AHF who were admitted to three tertiary care cardiac centers in the Delta region of Egypt within a period from April 2017 to May 2018, during which, data collection and short-term follow-up were done. Two of these centers have catheterization laboratory and they have primary percutaneous coronary intervention (PCI) programs. Very recently, one of them has arrhythmology department with electrophysiology service but still restricted to pacemaker implantation.

### Patient population

We included only patients with AHF whether de novo or acute on top of chronic HF. Patients with AHF were defined as those with progressive symptoms and signs of HF who required treatment with intravenous diuretics, inotropes, or vasodilators. We excluded patients who are less than 18 years of age, refused to give consent, and patients who were managed and discharged from the emergency department without hospital admission. For every patient, a standard case report form (CRF) has been filled out with an emphasis on clinical and demographic characteristics of included patients, course during hospital stay, and diagnostic and therapeutic options entertained prior to admission, during hospital stay and upon discharge. All CRFs were filled out by a cardiology resident with no interaction with the management plans of the treating physicians. Thirty-day and 90-day follow-up included all-cause mortality and re-admission rates were sought via telephone contact or pre-scheduled hospital visits. An ethical approval letter has been obtained from the ethics committees of the recruiting hospitals. Verbal informed consent has been obtained from each included patient or his substitute decision maker.

### Statistical analysis

Continuous variables were summarized using means or medians based on the normality; normally distributed variables were summarized using the mean and standard deviation (SD), while the non-normally distributed variables were summarized using the median. Categorical data were summarized as the frequency and percentages.

## Results

A total of 220 patients were enrolled in this registry, and 22 patients were lost for follow-up after 1 month from hospital discharge, while 8 patients were lost after 3 months. Overall, patients had a mean age of 58.79 years ± 14.36 and a median age of 61.50. As regard the gender, 65.5% were males. A review of medical history showed that 50.9% were known as heart failure patients before, 48.2% had hypertension, 42.7% had diabetes mellitus, 56.3% had smoking history, 28.2% had coronary artery disease ( 15.5% had prior PCI, and 2.7% had prior CABG), and 10.9% had rheumatic heart disease (4.5% had prior percutaneous or surgical valve interventions). For other comorbidities, 26.4% had atrial fibrillation, 6.4% had cerebro-vascular accidents (CVA) or transient ischemic attacks (TIA), 8.1% had bronchial asthma and COPD, and 10.9% had chronic kidney disease (CKD). None (0%) had a history of implantable cardioverter-defibrillator (ICD), cardiac resynchronization therapy (CRT) implantation, or permanent pacemaker (PPM) implantation. Table 1 summarizes the baseline demographic characteristics of our cohort.

**Table 1:** Clinical characteristics, investigations, and procedures in patients with acute decompensated heart failure

Overall, *n* = 220	
Demographics	
Male, %	65.5
Age in years, min–max	24.0–86.0
Age in years, mean ± SD.	58.79 ± 14.36
Age in years, median	61.50
Married, %	68.2
Disabled for working, %	67.3
Past medical history and risk factors	
Heart failure, %	50.9
Arterial hypertension, %	48.2
Diabetes mellitus, %	42.7
Coronary artery disease, %	28.2
Acute coronary syndrome, %	17.3
PCI, %	15.5
CABG, %	2.7
RHD, %	10.9
Valve repair or replacement, %	4.5
Atrial fibrillation, %	26.4
ICD, %	0.0
CRT, %	0.0
Cerebrovascular accident/transient ischemic attack (TIA), %	6.4
Peripheral arterial disease, %	0.9
Bronchial asthma/COPD, %	8.1
CKD, %	10.9
CKD on dialysis, %	1.8
Current/ex-smoker, %	56.3
Alcohol, %	0.0
Cancer chemotherapy, %	6.4
Symptoms and vital data upon admission	
Dyspnea, %	96.4
NYHA III/IV, %	90.6
PND/orthopnea, %	76
Lower limb swelling, %g	31.8
Palpitation, %	9.1
Lightheadedness/syncope, %	1.8
Chest pain, %	20
SBP (mmHg), mean ± SD	114.9 ± 33.45
SBP (mmHg), median	110.0
DBP (mmHg), mean ± SD	72.91 ± 19.79
DBP (mmHg), median	70.0
HR (b/m), mean ± SD	114.1 ± 23.94
HR(b/m), median	110.0
Oxygen saturation at room air (%), median	92.0
ECG findings	
Atrial fibrillation/flutter, %	35.5
Wide complex tachycardia, %	4.5
Ventricular fibrillation, %	4.5
LBBB, %	11.8
Early or evolved ECG criteria of STMI, %	17.3
Pathological Q waves, %	21.8
QRS width (ms), mean ± SD.	101.5 ± 20.64
QRS width (ms), median	100.0
Transthoracic echocardiography	
Patients with LVEF < 40%, %	62.6
Severe mitral incompetence, %	26.9
Coronary anatomy in 74 patients	
Significant left main disease, %	2.7
Significant single vessel disease, %	27
Significant double vessel disease, %	13.5
Significant three vessel disease, %	43.2
Non-significant CAD, %	2.7
Normal coronaries, %	10.8
Procedures during admission (*n* = 220)	
Coronary Reperfusion treatment, *n* (%)	32 (14.5)
Primary PCI strategy, *n* (%)	12, (37.5)
Pharmaco-invasive strategy, *n* (%)	4 (12.5)
Thrombolytic for STEMI, *n* (%)	16, (50)
Inotropic/vasopressor support, %	34.5
Assisted non-invasive ventilation, %	41.8
Invasive mechanical ventilation, %	12.7
Ventricular tachyarrhythmia event, %	7.3
Rise of serum creatinine > 0.3 mg/dl, %	38.2
Renal replacement therapy, %	1.8
Blood transfusion, %	5.5
Primary etiology of HF (*n* = 220)	
Ischemic heart disease, %	58.1
Valvular heart disease, %	16.3
Systemic hypertension related, %	9.1
Non-ischemic dilated cardiomyopathy, %	15.5
Others, %	0.9
HF exacerbating factors (*n* = 220)	
ACS, %	25.5
STEMI, %	17.3
NSTACS, %	8.2
Uncontrolled HTN, %	10.9
Infections, %	28.1
COPD exacerbation, %	1.8
Worsening renal function, %	9.1
Arrhythmia, %	11.8
Others, %	10.9
Non-compliance to HF medicines in ADHF patients (*n* = 112), %	19.6
Non-compliance to diet in ADHF patients (*n* = 112), %	23.2
All-cause mortality and re-hospitalizations	
In-hospital mortality, %	18.2
30 days mortality, %	20.7
90 days mortality, %	26
30 days re-hospitalization, %	10
90 days re-hospitalization, %	28.6

*PCI* percutaneous coronary intervention, *CABG* coronary artery bypass graft surgery, *RHD* rheumatic heart disease, *ICD* implantable cardioverter defibrillator device, *CRT* cardiac resynchronization therapy device, *TIA* transient ischemic attack, *COPD* chronic obstructive pulmonary disease, *CKD* chronic kidney disease, *NYHA* New York Heart Association Classification, *PND* paroxysmal nocturnal dyspnea, *SBP* systolic blood pressure, *DBP* diastolic blood pressure, *HR* heart rate, *WCT* wide complex tachycardia, *VF* ventricular fibrillation, *LBBB* left bundle branch block, *STEMI* ST elevation myocardial infarction, *LVEF* left ventricular ejection fraction, *CAD* coronary artery disease, *ACS* acute coronary syndrome, *NSTACS* non-ST elevation acute coronary syndrome, *HTN* hypertension, *HF* heart failure, *ADHF* acute decompensated heart failure

Dyspnea was reported by 96.4% as a main complaint, 49.1% were in New York Heart Association Classification (NYHA) class III, 41.5 % were in NYHA class IV, and 9.4% were in NYHA class II. Orthopnea and or paroxysmal nocturnal dyspnea (PND) were reported in 76%, 31.8% had lower limbs swelling, 9.1% had palpitation, 1.8% had syncope and/or pre-syncope, 20% had chest pain, and 9.1% reported other symptoms like fatigue, cough, hemoptysis, and fever. Median systolic blood pressure was 110 mmHg; median diastolic blood pressure was 70 mmHg; median heart rate was 110 beats/min; and median oxygen saturation was 94.0%.

Regarding the ECG at presentation, 35.5% had atrial fibrillation, 4.5% had wide complex tachycardia/VF, 17.3% fulfilled acute ST elevation myocardial infarction (STEMI) criteria, and 21.8% had pathological Q waves at presentation. The QRS width mean ± SD was 101.5 ± 20.64. LV EF estimation was calculated exclusively by using the M-mode method. The left ventricular ejection fraction (LVEF) mean ± SD was 38.69 ± 11.94, and the median was 35.0%. The percentage of patients with LVEF of less than 40% was 62.6%. A total of 33.6% of our study population has a known coronary anatomy by coronary angiography either before time of admission or during their hospital admission where 43.2% of them had evidence of multi-vessel disease (MVD), 20% had single-vessel disease, 10% had double vessel disease, and 2.7% had significant left main (LM) disease.

Coronary reperfusion has been done for 13.6% of our patients where 36.5% of them had primary PCI, 11.5% had pharmaco-invasive strategy, and 52% had thrombolytic therapy. Intravenous inotropic agents have been prescribed in 34.5% of patients, 41.8% had non-invasive respiratory ventilation (CPAP or BiPAP), 12.7 % had invasive endotracheal intubation and mechanical ventilation, 38.2% had worsening of serum creatinine, 1.8% had renal replacement therapy (hemodialysis/ultrafiltration), 5.5% had blood transfusion, and 7.3% had ventricular tachyarrhythmias (VT and/or VF).

**Fig. 1:**
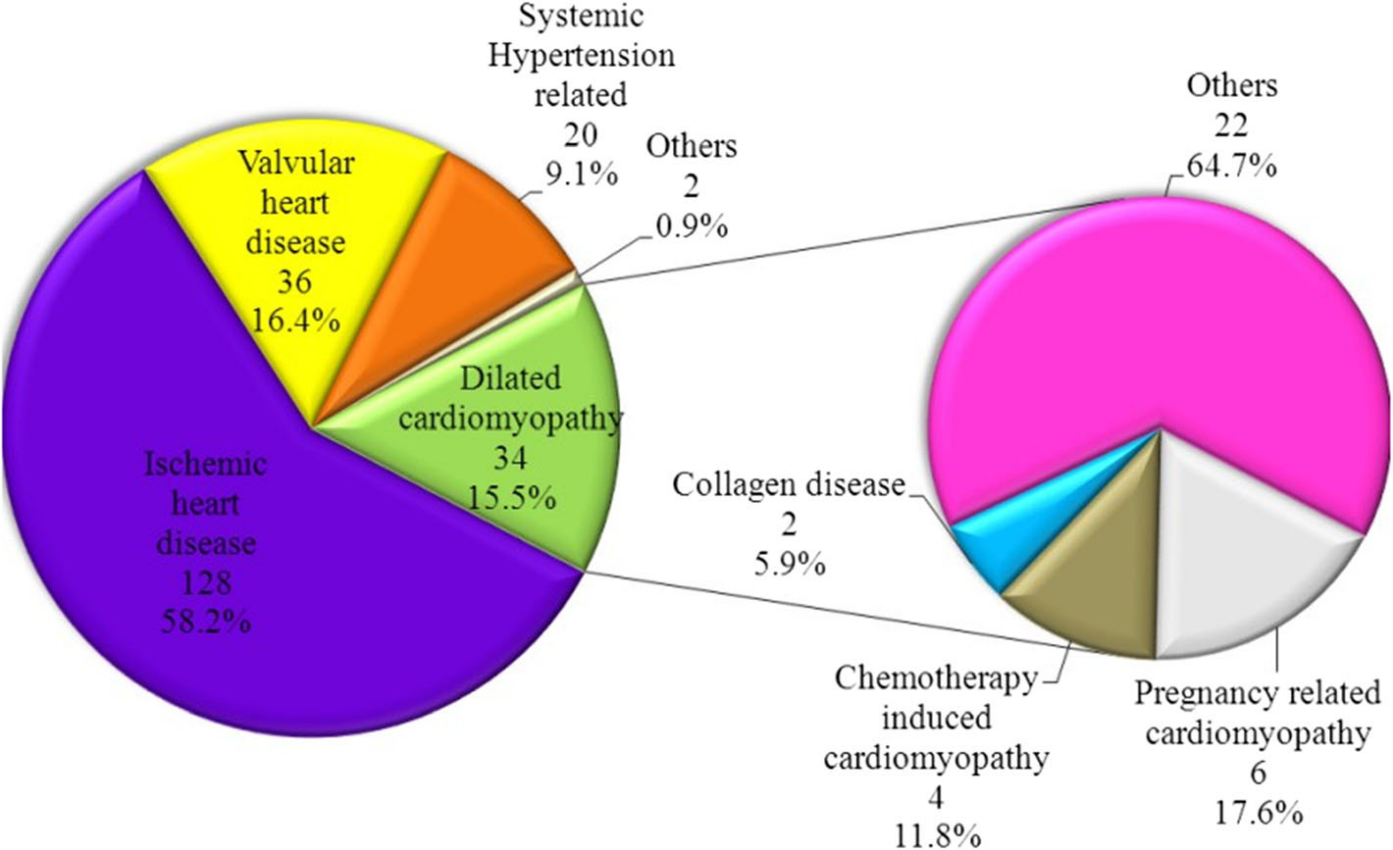
Etiology of acute heart failure in this patient cohort (*n* = 220)

**Fig. 2:**
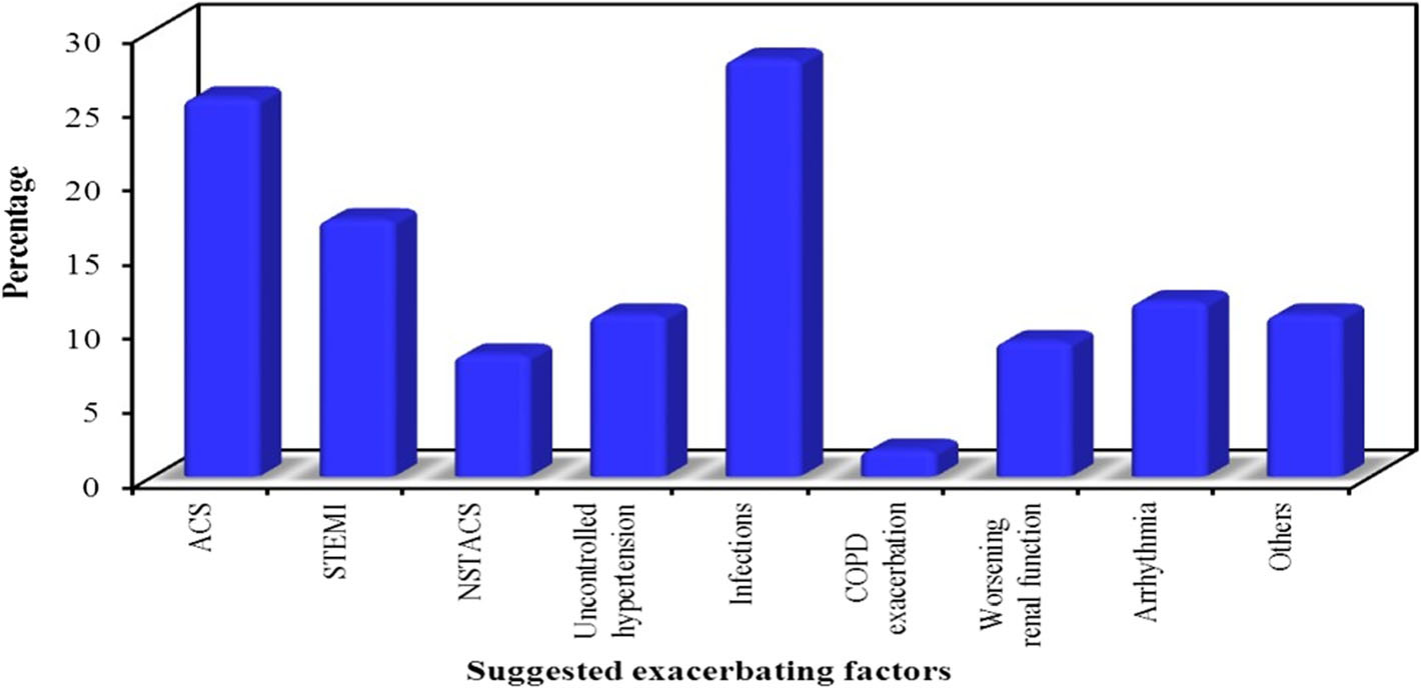
Exacerbating factors for acute decompensation in this heart failure patient cohort (*n* = 220)

Ischemic heart disease has been suggested as the underlying primary etiology in 58.1% of patients, valvular heart disease in 16.3%, dilated non-ischemic cardiomyopathy in 15.5%, and systemic arterial hypertension in 9.1% as shown in Fig. 1. ACS including (STEMI or NSATCS) as an exacerbating factor was identified in 25.5% of cases, infections (respiratory or non-respiratory) in 28.1%, uncontrolled hypertension in 10.9%, arrhythmias in 11.8%, worsening renal function in 9.1%, and COPD exacerbation in 1.8% as shown in Fig. 2. In acute decompensated heart failure (ADHF) patients’ group, non-compliance to diet was an exacerbating factor in 23.2% of cases while it was 19.6% for non-compliance to medications.

In-hospital mortality rate was estimated to be 18.2%. Mortality rate after 1 month was estimated to be 2.5% and 5.3% after 3 months. Re-admission rate was 10% after 1 month and 18.6% after 3 months follow-up (Fig. 2).

As regards to oral medications before time of admission, 24.5% were on beta-blockers, 20.9% were on ACE inhibitors, 10.9% were on ARBs, 21.8% were on MRAs, 22.7% were on digoxin, 54.5% were oral diuretics, 9.1% were on ivabradine, 21.8% were on nitrates, 5.5% were on amiodarone, 1.8% were on non-dihydropyridine calcium channel blockers (CCB), 3.6% were on dihydropyridine CCB, 24.5% were on statins, 24.5% were on warfarin, 32.7% were on aspirin, 13.6% were on P2Y12 receptor inhibitors, and 6.4% were on trimetazidine. While upon discharge, 50% have been prescribed beta-blockers, 63.3% ACE inhibitors, 13.3% ARBs, 52.2% MRAs, 10% digoxin, 92.2% diuretics, 17.7% ivabradine, 20% nitrates, 14.4% amiodarone, 1.1% non-dihydropyridine CCB, 5.5% dihydropyridine CCB, 56.6% statins, 38.3% warfarin, 48.8% aspirin, 28.8% P2Y12 receptor inhibitors, and 1.1% trimetazidine as shown in Table 2.

**Table 2:** Oral medications before admission and upon discharge

	Pre-admission, *n* = 220	Upon discharge, *n* = 180
Beta-blockers, %	24.5	50
ACE inhibitors, %	20.9	63.3
ARBs, %	10.9	13.3
MRAs, %	21.8	52.2
Digoxin, %	22.7	10.0
Diuretics, %	54.5	92.2
Ivabradine, %	9.1	17.7
Nitrates, %	21.8	20.0
Amiodarone, %	5.5	14.4
Non-dihydropyridine CCBs	1.8	1.1
Dihydropyridine CCBs, %	3.6	5.5
Statins, %	24.5	56.6
Warfarin, %	24.5	38.8
Aspirin, %	32.7	48.8
P2Y12 receptor inhibitor, %	13.6	28.8

*ACE* angiotensin converting enzyme, *ARB* angiotensin receptor blocker, *MRA* mineralocorticoid receptor antagonist, *CCB* calcium channel blocker

## Discussion

Heart failure (HF) is a major cause of morbidity and mortality worldwide and has significant negative impacts on quality of life, healthcare costs, and longevity [10]. Registries have been carried out for better understanding of the epidemiology, clinical presentation, management, and outcome of heart failure patients. Most of the data came from Western countries till the last few years when at least three well-designed registers have enriched the literature with data from Gulf region and Egypt [7–9, 11]. In our registry, we reported demographics, baseline clinical presentation, management, and short-term outcomes of 220 patients with acute heart failure, and we discussed our results in comparison to other data from the western and Gulf regions.

The average age of presentation in our patients was approximately one decade younger relative to patients in the Western countries but was very comparable to the age of patients in registries from the Middle East region [4, 7, 8]. This is probably explained by the high rates of smoking, diabetes mellitus, and hypertension. The high rates of CAD risk factors might have been associated with the presence of ischemic etiology in more than half of our acute heart failure patients.

Atrial fibrillation on ECG was found in approximately one-third of our patients, while its prevalence was lower in ADHERE (20%) [10], HEARTS (17%) [7], Gulf CARE (14%) [8], and Hassanein et al.’s cohort (24.3%) [9, 11]. Higher prevalence of atrial fibrillation in our patients might go with higher prevalence of patients with NYHA class IV which in part reflects the severity of heart failure. The prevalence of atrial fibrillation in patients with HF varies from < 10% to 50% in different registries [12].

LVEF estimation via TTE was done exclusively by M-mode method; however, recent 2016 ESC guidelines have recommended for measurement of LVEF, the modified biplane Simpson’s. This method relies on accurate tracing of endocardial borders [13]. More than one-third of our patients had LVEF of at least 40%, while they were 27.1%, 31.0%, and > 22.0% in HEARTS [7], Gulf CARE [8] and Hassanein et al.’s cohort, respectively [9, 11].

None of our patients has undergone natriuretic peptide (NP) testing, and this was attributed mainly to unavailability of the test, lack of physician orientation of its role in AHF management, and the high costs of the test. Measurement of NPs is recommended in patients presented with acute dyspnea and confusion about its etiology being cardiac versus other non-cardiac and also to assess the prognosis and to guide treatment in both patients with acute and chronic heart failure [13, 14].

We have 38 patients presented with STEMI and 18 patients presented with non-STEMI; however, reperfusion treatments have been offered to only 32 patients which represent slightly more than half of patients who presented with acute coronary syndrome (ACS). Primary PCI strategies are generally underutilized in our Delta hospitals especially in non-STIMI patients, and this is attributed mainly to economic issues. Non-STEMI 2015 ESC guidelines recommend an immediate invasive strategy with intent to perform revascularization, irrespective of ECG or biomarker findings in patients with at least one very high-risk criterion (hemodynamic instability, cardiogenic shock, acute heart failure AHF, life-threatening arrhythmia, cardiac arrest, recurrent chest pain refractory to medical treatment, or dynamic ST changes particularly transient ST elevation) [15]. The Stent for Life Initiative project largely succeeded to offer an equal access to primary PCI for patients with STEMI, while efforts are still on to expand that for patients presented with very high risk NSTEMI. Fibrinolytic therapy is an important reperfusion strategy in settings where primary PCI cannot be offered in a timely manner [16].

None of our patients received device therapy (ICD, CRT, or both) or even offered these therapies despite we had 40 patients with known LVEF less than 35%, 4 patients with QRS width (ms) ≥ 150, and 8 patients with QRS width (ms) ≥ 130. Underutilization of device therapy is largely related to socioeconomic issues, unavailability of the service and trained teams, and lack of physician orientation. According to 2016 HF ESC guidelines, ICD is recommended to reduce the risk of sudden death and all-cause mortality in patients with symptomatic HF (NYHA classes II–III) and an LVEF ≤ 35% despite ≥ 3 months of optimal medical therapy (OMT), provided they are expected to survive substantially longer than 1 year with good functional status and they have ischemic heart disease “unless they had MI in previous 40 days” and dilated cardiomyopathy. Also, it is recommended as a secondary prevention measure to reduce the risk of sudden death and all-cause mortality in patients who have recovered from a ventricular arrhythmia causing hemodynamic instability, and who are expected to survive for > 1 year with good functional status. Also, CRT is recommended for symptomatic patients with HF in sinus rhythm with a QRS duration ≥ 130 ms and LBBB morphology and with LVEF ≤ 35% should be considered for those in sinus rhythm with a QRS duration ≥ 150 ms and non-LBBB morphology and with LVEF ≤ 35% and may be considered for those in sinus rhythm with a QRS duration ≥ 130 ms and non-LBBB morphology and with LVEF ≤ 35% despite OMT in order to improve symptoms and reduce morbidity and mortality [13].

Before the time of admission, less than one-quarter of our patients were on beta-blockers, less than one-third on angiotensin-converting enzyme inhibitors (ACEIs)/ARBs, and about one-fifth were on MRAs. Upon discharge, the figures improved but still below the ideal with half of them were put on beta-blockers, about 3 quarters on ACEIs/ARBs, and more than one half on MRAs. There is relatively large gap in prescribing guideline-directed medical therapy (GDMT) particularly beta-blockers (BB) when we compare our data with the regional and international registries which might be explained, at least in part, by limited hospital resources and lack of heart failure programs or protocols in these hospitals.

### Study limitations

There were some limitations of this registry; most of them related to the limited sample size and limited geographical representation. Some cases were not selected on a consecutive basis due to the lack of hospitals’ database and unavailability of a person responsible for collecting the data during the course of admission of some patients. Small sample is another important limitation which might make this registry less representative for heart failure in Egypt. Absence of hospital database systems for patients’ admission and follow-up made it difficult for us to collect the data, and some cases were missed during follow-up phases. Natriuretic peptide testing was not included in the diagnosis of AHF which was based mainly on clinical examination and TTE findings.

## Conclusions

There is a clear gap in the management of patients with heart failure in the Delta region of Egypt with confirmed very low use of NPs in the diagnosis and prognostication of heart failure, under-utilization of heart failure device therapy, low rate of coronary intervention, and under-prescription of guideline-directed medical therapies particularly beta-blockers. Limited resources and the lack of heart failure management programs are the main causes of this gap. We are planning to have a second phase of this registry with a bigger sample size and more hospitals representing the Delta region in Egypt.

## Data Availability

The datasets used and analyzed during the current study are available from the corresponding author on reasonable request.

## Abbreviations

ACEI: Angiotensin-converting enzyme inhibitor
ADHF: Acute decompensated heart failure
AHF: Acute heart failure
CKD: Chronic kidney disease
COPD: Chronic obstructive pulmonary disease
CRF: Case report form
CRT: Cardiac resynchronization therapy
CVA: Cerebro-vascular accident
GDMT: Guidelines directed medical therapy
HF: Heart failure
ICD: Implantable cardioverter-defibrillator
LA: Left atrium
LVEF: Left ventricular ejection fraction
MVD: Multi-vessel disease
NPs: Natriuretic peptides
PPM: Permanent pacemaker
TIA: Transient ischemic attacks
